# Vesicoureteral Reflux and Innate Immune System: Physiology, Physiopathology, and Clinical Aspects

**DOI:** 10.3390/jcm12062380

**Published:** 2023-03-19

**Authors:** Marius-Cosmin Colceriu, Paul Luchian Aldea, Andreea-Liana Răchișan, Simona Clichici, Alexandra Sevastre-Berghian, Teodora Mocan

**Affiliations:** 1 Department of Functional Biosciences, Discipline of Physiology, Iuliu Haţieganu University of Medicine and Pharmacy, 400006 Cluj-Napoca, Romania; 2 Department of Community Medicine, Discipline of Public Health and Management, Iuliu Haţieganu University of Medicine and Pharmacy, 400006 Cluj-Napoca, Romania; 3 Department of Mother and Child, Discipline of Pediatrics II, Iuliu Haţieganu University of Medicine and Pharmacy, 400006 Cluj-Napoca, Romania; 4 Nanomedicine Department, Regional Institute of Gastroenterology and Hepatology, 400158 Cluj-Napoca, Romania

**Keywords:** vesicoureteral reflux, urinary tract infection, innate immunity, cytokines, antimicrobial peptides

## Abstract

Vesicoureteral reflux represents one of the most concerning topics in pediatric nephrology due to its frequency, clinical expression with the potential to evolve into chronic kidney disease, and last but not least, its socio-economic implications. The presence of vesicoureteral reflux, the occurrence of urinary tract infections, and the development of reflux nephropathy, hypertension, chronic kidney disease, and finally, end-stage renal disease represent a progressive spectrum of a single physiopathological condition. For the proper management of these patients with the best clinical outcomes, and in an attempt to prevent the spread of uropathogens’ resistance to antibacterial therapy, we must better understand the physiopathology of urinary tract infections in patients with vesicoureteral reflux, and at the same time, we should acknowledge the implication and response of the innate immune system in this progressive pathological condition. The present paper focuses on theoretical aspects regarding the physiopathology of vesicoureteral reflux and the interconditionality between urinary tract infections and the innate immune system. In addition, we detailed aspects regarding cytokines, interleukins, antimicrobial peptides, and proteins involved in the innate immune response as well as their implications in the physiopathology of reflux nephropathy. New directions of study should focus on using these innate immune system effectors as diagnostic and therapeutic tools in renal pathology.

## 1. Introduction

One of the most concerning topics in pediatric nephrology is vesicoureteral reflux, due to its frequency and potential to evolve into chronic kidney disease (CKD) [1].

Vesicoureteral reflux (VUR) is defined as retrograde urine flow from the urinary bladder to the ureter and urinary collecting system of the kidneys. This pathologic condition is supposed to be present in 1% to 2% of all children (up to 20% of infants with a family history of VUR), which makes VUR one of the most common medical conditions that lead to hospitalization, especially in young children [1,2].

It is considered that up to one-third of children with VUR will develop at least one febrile urinary tract infection (UTI). Frequently, these children develop recurrent pyelonephritis, which can lead to renal scarring, also known as reflux nephropathy (RN). In some patients (up to 25%), reflux nephropathy can progress to end-stage CKD requiring renal replacement therapy such as dialysis or kidney transplant. About 6% of children and young adults with end-stage renal disease (ESRD) have VUR as an etiology [1,3,4].

Even though the common belief has been that VUR predisposes the patient to febrile urinary tract infections, and both together can cause reflux nephropathy, which can lead to hypertension and end-stage renal disease, lately, there are some controversies regarding the role of UTI in the pathophysiology of reflux nephropathy. This is because there are some clinical observations according to which RN can occur even in the absence of UTIs, especially in male patients with high-grade VUR with antenatal onset [5,6,7].

Current research efforts related to VUR are focusing on a better understanding of genetic mechanisms and finding new biological markers associated with an increased risk of developing chronic kidney disease. Another benefit of the identification of new molecules involved in the pathophysiology of VUR is related to the management and treatment of these patients, to achieve the best outcome for each patient [4,5].

## 2. Pathophysiology of VUR

Based on pathogenesis, VUR is classified as primary or secondary. The most common type is primary VUR, which occurs as a congenital malfunction of the ureterovesical junction. With patient growth, many of these cases evolve to spontaneous resolution, especially low-grade VUR. On the other hand, VUR may be secondary to abnormally high pressure in the urinary bladder, which forces the urine to flow back into the ureters despite the physiological resistance of the vesicoureteral junction. Such diseases that produce secondary VUR are neurogenic bladder or posterior urethral valves [2,8,9].

From the bladder, either infected or non-infected urine can enter the collecting system of the kidneys, causing inflammation that can lead to renal scarring, proteinuria, hypertension, and impaired renal function. The presence of infected urine in kidneys activates a local immune response, which in the case of persistent VUR may lead to chronic inflammation and fibrogenesis, followed by parenchymal healing with scars. Anatomical reshaping of the renal papillae perpetuates further intrarenal reflux, initiating a vicious cycle that leads to the formation of new scars. In the case of non-infected urine reflux, there is no evidence of renal scar development. This observation applies to low-grade VUR in the absence of urinary infections. In children with grade IV or V of RVU, there may be diffuse parenchymal lesions, even if there is no history of urinary tract infections, which results in abnormal renal development. Another clinical observation is that patients with low-grade VUR who have an underlying pathological condition that causes abnormally increased detrusor pressure may develop reflux nephropathy in the absence of infections. Renal scar development in the absence of infections is explained by the effect of the high pressure of non-infected urine on the renal parenchyma. This leads to local ischemia with the release of proinflammatory cytokines, toxic metabolites, and reactive oxygen species. In this way, local inflammation occurs, and a process of architectural remodeling and fibrosis is initiated, especially under the action of metalloproteinases, leading to renal scar development. In addition to this tubulointerstitial inflammatory process, the high pressure of non-infected urine determines glomerular injury with secondary proteinuria, which maintains the local inflammatory response. Moreover, an altered synthesis of prostaglandins has been described [1,2,3,10,11,12,13].

To sum up, renal scarring can appear in patients with UTI without VUR or in patients with VUR without UTI, although it was previously believed that the coexistence of the two was mandatory. RN due to VUR alone is especially seen in newborns, as a congenital form of RN or, as mentioned above, in conditions associated with high intravesical pressure. The congenital form of RN occurs mostly in males, whereas RN due to UTIs occurs more frequently in females [10,11,12,13].

The incidence of RN is directly proportional to the age at the diagnosis of VUR. Some studies report an incidence of RN of 10% in newborns, 26% in patients under 8 years, 47% in patients older than 8 years, and 94% in adult patients [10,13,14].

Microalbuminuria, which indicates early stages of glomerular impairment, and proteinuria are determined by immunologic injury, mesangial dysfunction, macromolecular trapping, hyperfiltration, and hypertension. Urine excretion of proteins suggests glomerular or tubular damage and is correlated with the severity of RN and progression to CKD [9,10,13,15].

Renal parenchymal lesions resulting from intrarenal urine reflux predispose the patient to hypertension by renin-angiotensin system activation, representing one of the most common causes of hypertension in children. In one study on the pediatric population with new-onset hypertension and no other pathologic condition, the results showed that 21% of children had renal scars in the presence of VUR. On the other hand, hypertension occurs in 10–30% of patients with RN [4,9,10,13].

The endpoint of these structural and functional changes is represented by the progression to CKD and ultimately the development of ESRD, which occurs in about 4% of patients with VUR (7–17% of patients with RN) [10,14].

In conclusion, the immune response to infectious aggression and subsequent triggering of the inflammatory process is responsible for initiating the cascade of pathophysiological processes that leads to renal parenchyma damage. However, in certain conditions, as mentioned above, VUR alone, due to sterile pressure effects, may be able to produce tissue remodeling with fibrosis [3,11].

Knowing the immunological mechanisms that take place in the urinary tract, we can better understand the pathophysiology of reflux nephropathy, which can guide us to better management of patients with vesicoureteral reflux.

## 3. Urinary Tract Infections and Innate Immune System

**Figure 1:**
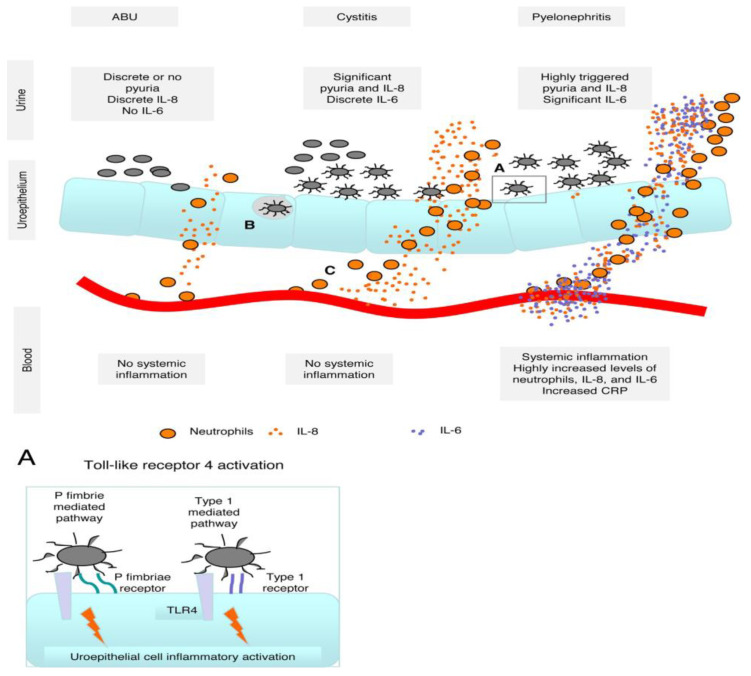
Schematic figure outlining bacterial challenge and activation of the host response in urinary tract infections. (A) In asymptomatic bacteriuria, there is no or only inefficient activation of the uroepithelium. In symptomatic urinary tract infection, bacterial contact with the uroepithelium is mediated by P or type 1 fimbriae and its receptors on the uroepithelium. Toll-like receptor 4 recognizes the Gram-negative uropathogens, the uroepithelial cell is activated, and inflammatory mediators (interleukin (IL)-6 and IL-8) are produced. (B) In recurrent cystitis, intracellular bacterial fabrics have been suggested. (C) Neutrophils from the circulation transmigrate, guided by expressed IL-8 receptors (CXCR), by following the concentration gradient of IL-8 to the place of infection to combat the bacteria by phagocytosis. ABU = asymptomatic bacteriuria; CRP = C-reactive protein; IL = interleukin; TLR4 = toll-like receptor 4; UTI = urinary tract infection. Reprinted/adapted with permission from [18]. 2023, Béla Köves, Björn Wullt.

Normally, the urinary tract remains sterile. However, when bacterial agents end up colonizing urine and the urothelium, conditions such as asymptomatic bacteriuria, cystitis with local inflammation, and pyelonephritis with a systemic inflammatory response may develop (Figure 1) [16,17].

Another important aspect that needs to be taken into consideration when we discuss UTIs is the urinary microbiome. The urobiome and its implications in the development of UTIs have been intensively studied in recent years. Even though the commensal microorganisms in the urinary tract are less abundant than in other anatomical sites, they play a crucial role in maintaining local homeostasis and preventing infection development. The occurrence of local dysbiosis has been proven to be implicated in several pathological conditions such as UTIs, urolithiasis, bladder dysfunction, or renal malignancies. In certain conditions that lead to urinary dysbiosis, dormant intracellular uropathogens, such as *E. coli*, can be activated. This can influence the local immune response, with IL-1 receptor activation and apoptosis induction, leading to uroepithelial injury and UTI recurrences. Considering the urobiome implication in local immune expression and responses to antibiotic treatment, it could be an important element involved in the physiopathology of VUR, which can be influenced in order to prevent the evolution to RN. One way of doing this is with probiotics therapy in addition to the use of rational antibiotics [19,20,21,22].

**Figure 2:**
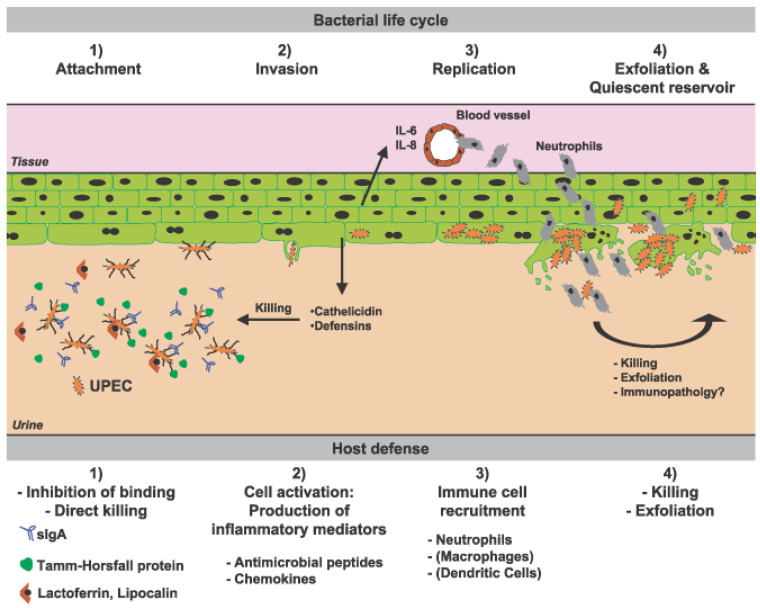
Multilayered effector mechanisms to combat urinary tract infections (UTI). Soluble factors such as lactoferrin, Tamm–Horsfall protein (THP), or secretory IgA inhibit attachment of uropathogenic *E. coli* (UPEC) to the epithelium. Despite these defense mechanisms, some bacteria are able to attach and invade the epithelium, which in turn releases antimicrobial molecules such as cathelicidin or defensins. Moreover, the uroepithelium produces interleukin (IL)-6 and IL-8 to attract additional immunocompetent cells such as neutrophils, which help to eliminate the pathogens [25].

It is observed that children with several pathological conditions associated with severe immunodeficiencies, such as a deficiency of primary antibodies or severe combined immunodeficiency syndrome, rarely develop urinary tract infections. Usually, when these patients develop urinary infections, a urinary tract abnormality is suspected. This indicates that the integrity of the urothelium with a normal local innate immune response and normal urine flow is the cornerstone of antibacterial defense (Figure 2) [23,24].

As such, when a pathogen reaches the urothelium, it interacts with toll-like receptors (TLRs) that activate several intracellular signaling pathways, triggering the innate immune system. This immediate response involves the production of cytokines and chemokines, generating local inflammation that can evolve to full recovery or, if persistent or recurrent, may initiate fibrogenesis with renal scar development [23,26].

After TLRs’ activation by lipopolysaccharides on the bacterial surface, several soluble factors, such as uromodulin, cathelicidin, pentraxins, or neutrophil gelatinase-associated lipocalin (NGAL), are released in an attempt to eliminate the uropathogens [22,27].

The innate immune system comprises effectors such as cellular elements (epithelial cells, dendritic cells, natural killer cells), antimicrobial peptides (AMPs), proteins, cytokines, chemokines, and reactive oxygen and nitrogen species [23,28].

### 3.1. Toll-Like Receptors

After bacterial colonization of the urinary tract and the attachment of uropathogens to urothelial cells, the innate immune response is initiated. The first step is the recognition of uropathogens as external and possibly harmful entities, different from self-structures. This is possible by identifying certain structures on the surface of bacterial cells by pattern recognition receptors (PRRs). The latter are generally located on the surface of cells and have the role of triggering the innate immune response by activating certain intracellular signaling mechanisms. PRRs are present on the surface of epithelial cells of the urinary tract and also on immune active cells such as macrophages or dendritic cells [26,28,29].

One of the most studied families of PRRs, with a crucial role in triggering an innate immune response in the urinary system, is the family of toll-like receptors (TLRs). These receptors are localized on the surface of leukocytes and epithelial cells of the urinary tract. Their structure consists of the presence of three domains: a large extracellular domain rich in leucine, a transmembrane domain, and an intracellular domain. The first domain has a role in the recognition of uropathogenic structures present on cell surfaces. The binding of certain components of the bacterial structure will cause a conformational change in the TLRs’ structure, which will determine the interaction of the intracellular domain with certain cytoplasmic proteins. This will lead to the activation of intracellular signaling mechanisms with the production of proinflammatory cytokines, interferons, and chemokines, consequently triggering the local innate immune response [30,31].

Thus far, thirteen receptors have been identified from the TLR family in humans (TLR1-TLR13), and nine receptors have been identified in murine models. Of these, the most studied receptors with a role in the pathophysiology of urinary tract infections are TLR2, TLR4, and TLR5 in humans and TLR 11 in murine models. TLR4, in addition to being the most studied receptor, is also the main receptor that binds lipopolysaccharides from Gram-negative bacterial cell walls. Thus, through the mechanism described above, the local innate immune response is triggered, resulting in the recruitment of neutrophils, the release of AMPs, and subsequent bacterial clearance. The presence of TLR4 on the surface of the urothelium and immune cells is essential in order to combat the development of urinary tract infections. Together with TLR4, TLR5, which is expressed in renal collecting ducts, plays a key role in *E. coli* clearance in pyelonephritis [26,32,33].

By activating the immune response and implicitly the inflammatory reaction, TLRs contribute to renal parenchymal damage mechanisms. To counterbalance this activity, there are several innate regulatory mechanisms, such as co-receptors, intracellular trafficking, or negative regulators. Some studies have shown that individuals with low TLR expression are predisposed to bacterial colonization and asymptomatic bacteriuria. On the other hand, low TLR expression leads to the absence of an inflammatory response, which could be a protective factor for renal tissue [28,30].

### 3.2. Transcription Factors

**Figure 3:**
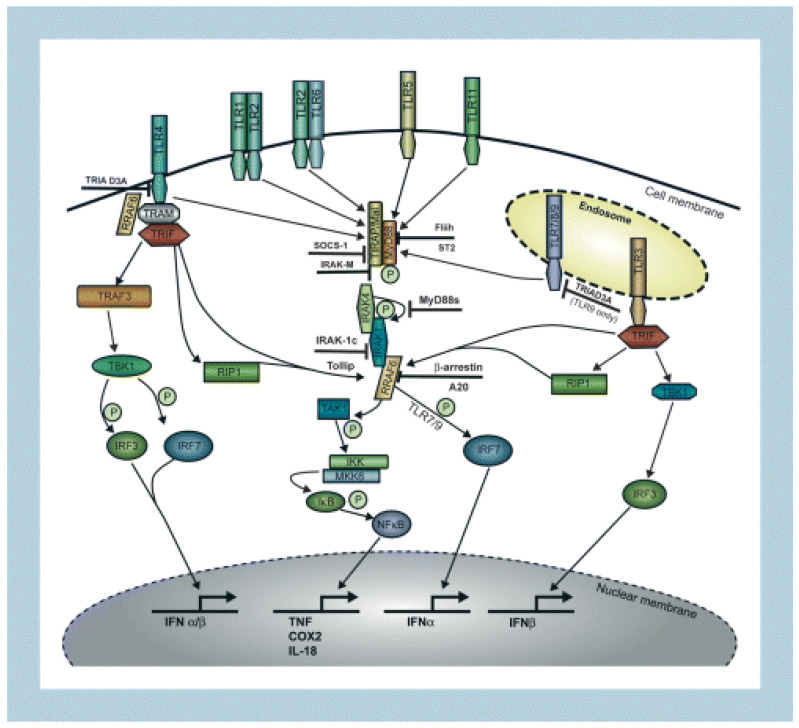
Toll-like receptor (TLR) signaling. Ligand binding to their cognate TLRs induces two signaling pathways, the MyD88-dependent and the MyD88-independent pathway. Four adaptor proteins (MyD88, TIRAP (Mal), TRIF (TICAM-1), and TRAM (TICAM-2)) selectively activate these two signaling pathways, leading either to the production of proinflammatory cytokines or type I interferons (IFNs) [34].

As mentioned above, TLRs’ activation leads to the triggering of intracellular signaling mechanisms that have the role of modulating the innate immune response. There are two pathways that are described. The first is MyD88 (myeloid differentiation primary response 88) dependent, in which the transcription factor is represented by nuclear factor kappa B (NF-kB). The second is MyD88 independent, in which the transcription factors are represented by interferon regulatory factors 3 and 7 (IRF3 and IRF7) (Figure 3) [28,30].

MyD88 is a protein with a role in information transfer between immune cells. It acts as an adapter, internalizing information from extracellular structures to intracellular proteins. Thus, MYD88 makes contact with the intracellular domain of TLR4 and transfers the signal to NF-kB. The latter is thus activated and stimulates the transcription of certain pro-inflammatory genes involved in both innate and adaptative immune responses. In addition, NF-kB is involved in the activation of inflammasomes and the regulation of T cells’ differentiation and activity [35,36].

In the MyD88-independent pathway of intracellular signaling, the information transfer from TLR4 to transcription factors is mediated by adaptor protein Toll/IL-1 receptor (TIR) domain-containing adaptor-inducing IFN-ß (TRIF), leading to IRF3 and IRF7 activation [28,36].

IRF3 is an important mediator in the regulation of the antimicrobial response during urinary tract infections. In murine models that lack IRF3, increased susceptibility to intensive bacterial colonization, the development of severe pyelonephritis with urosepsis, and massive tissue damage have been observed. IRF7, on the other hand, modulates the inflammatory response during a UTI. In murine models that lack IRF7, lower neutrophil accumulation in urine and urothelium have been observed, as well as lower bacterial burden. Thus, it can be concluded that IRF7 is responsible for a hyperinflammatory response during UTIs. Therefore, for an adequate immune response, a balanced expression of IRF3 and IRF7 is needed. It is supposed that genetic expression of IRF3 may influence susceptibility to urinary tract infections. It has been observed that individuals with lower IRF3 expression present recurrent acute pyelonephritis. In contrast, individuals with lower IRF expression present asymptomatic bacteriuria, being protected from recurrent acute pyelonephritis. Studies have shown that IRF7 suppression has a similar outcome to antibiotic therapy in order to prevent renal abscess formation, which is why it could be a target of immunotherapy [31,35,37,38].

Finally, regardless of the intracellular signaling pathway, cytokines, chemokines, interferons, and AMPs will be synthesized and released in the urinary stream, leading to an inflammatory response and bacterial clearance [35,36].

### 3.3. Cytokines and Chemokines

Cytokines represent a broad category of small secreted proteins, usually up to 80 kDa, with a role in cell-to-cell interaction and communication. Depending on the cells upon which they act, cytokines may have autocrine activity (i.e., act on the same cells that secrete them), paracrine activity (i.e., act on neighboring cells), or endocrine activity (i.e., act on distant cells). Cytokines are produced by a wide variety of cells. Depending on the cell by which they are produced, cytokines have different names. Therefore, if they are produced by lymphocytes, they are known as lymphokines; if they are produced by monocytes, they are known as monokines; if they are produced by leukocytes and act on other leukocytes, they are known as interleukins; if they have chemotactic properties, they are known as chemokines; if they are involved in antiviral responses, they are known as interferons; and if they stimulate the growth of different cells, they are known as colony-stimulating factors. As they are involved in communication between immune cells, cytokines are important modulatory agents in innate and adaptive immune responses and also in inflammatory reactions, being able to manifest either pro- or anti-inflammatory activity. The same cytokine may be produced by different types of immune cells and can exert action on different cells, this property being known as pleiotropy. Different cytokines can exert the same activity on immune cells and can also stimulate other cytokines’ production, resulting in an amplified immune response. Their actions can be either antagonistic or synergistic [28,39,40,41,42].

Two important cytokines involved in the immune response during urinary tract infections, and also with possible implications in the pathophysiology of reflux nephropathy, are IL-6 and IL-8 [26,43,44].

IL-6 is an extensively studied cytokine with proinflammatory activity. It is expressed by a wide variety of cells such as fibroblasts, macrophages, activated T and B cells, mesangial and tubular cells in the kidneys, and endothelial cells. As mentioned above, after TLRs’ stimulation and the activation of intracellular signaling mechanisms, IL-6 is promptly released into the urinary stream as a response to uropathogens’ colonization, with this process taking place in the early stages of the innate immune response. IL-6 is a pyrogen mediator of inflammation response, which is involved in fever development and also in the induction of acute phase reaction, including liver production of the C-reactive protein and other acute phase proteins. In addition, IL-6 plays an important role in the activation and differentiation of T and B cells during the inflammatory response. It also induces AMP expression, thus facilitating bacterial clearance. Other mechanisms by which IL-6 is involved in the response to uropathogens’ aggression are the stimulation of monocyte proliferation and the alteration of iron metabolism, thereby preventing bacterial growth and multiplication [28,45,46,47,48].

In individuals with reflux nephropathy, immunohistochemical studies have shown that the major site of IL-6 synthesis is represented by renal tubular cells located near areas of fibrosis. This observation brings into question the role of IL-6 in reflux nephropathy pathophysiology [45,49].

The studies performed on animal models with a lack of IL-6 expression have shown that in individuals with IL-6 deficiency, bacterial colonization is more intense, with uropathogens being able to escape the innate immune response and thus promote chronic inflammation. These individuals also present more severe UTIs, more pronounced structural alterations of the renal parenchyma, and ultimately, higher mortality rates [50,51].

Several studies have been performed in order to investigate the role of IL-6 in febrile UTIs, VUR, and RN. Such a study conducted by Sheu et al. showed that there were no significant differences in urine and serum concentrations of IL-6 between children with febrile UTI with VUR and children with febrile UTI without VUR. Another study conducted by Gokce et al. revealed that in pediatric patients without recent UTIs, individuals with VUR have higher levels of urine IL-6. Other studies performed by Jutley et al. and Sheu et al. have shown that there were higher concentrations of serum IL-6 in patients with VUR or RN, and that elevated serum and urine IL-6 concentrations are correlated with a higher risk of renal scar development. Wang et al. conducted a study that revealed that children with reflux nephropathy present higher concentrations of IL-6 in urine, with these concentrations correlating with the severity of renal scars [46,48,49,50,51].

From all of the above, we cannot draw categorical conclusions regarding the biomarker role of IL-6 in the studied pathologies. Explanations for the contradictory results could be the presence of a recent acute inflammatory process that influenced serum and urinary IL-6 concentrations in patients with VUR and RN or the use of therapeutic interventions, such as chemoprophylaxis or surgical correction of VUR. Another explanation could be the assays used in the studies and the differences in the detection limits [17,51].

However, the results obtained by most studies support the idea of using IL-6 as a biomarker of UTI severity, as it is able to differentiate acute cystitis from acute pyelonephritis. In addition, IL-6 seems to be a potential marker in detecting renal scars and chronic renal injury. Nevertheless, further studies are needed [51].

IL-8 has the same pathway of production as that described above regarding IL-6. Known also as CXCL8, IL-8 is a protein with chemotactic activity during the immune response. It has the ability to attract neutrophils and lymphocytes at the site of the inflammatory process. After the interaction of uropathogens with TLRs and the triggering of the innate immune response, IL-8 is produced by renal mesangial and epithelial cells and also by circulating and recruited immune cells. The main IL-8 role is to facilitate neutrophil migration from blood to the urinary stream through the urothelium. Thereby, IL-8 is responsible for the presence of leukocytes in the infected urine, which is known as pyuria. There are two receptors for IL-8 on the surface of urothelial cells: CXCR1 and CXCR2. The first has a higher affinity in binding IL-8 and is essential for neutrophil migration across infected urothelial cells. The expression of CXCR1 increases in UTIs. Experimental studies have shown that a lack of the CXCR1 leads to the inability to eliminate the uropathogens, renal abscess, bacteremia, and renal scar formation. More than this, children with susceptibility to recurrent acute pyelonephritis have lower expression of CXCR1 in comparison with healthy individuals [26,28,51,52].

The studies published so far have presented results according to which IL-8 concentrations are higher in the urine of patients with acute pyelonephritis, VUR, and renal scars. It is supposed that elevated urinary IL-8 is involved in reflux nephropathy development in patients with VUR even in the absence of UTIs. This is why IL-8 may be useful as a biomarker of VUR diagnosis and also as a prognostic biomarker regarding the evolution toward RN [49,51,53].

Higher urinary IL-8 concentrations have been also detected in patients with congenital anomalies of the kidney and urinary tract, which are associated with impaired renal function. In these cases, IL-8 levels correlate with eGFR. These observations support the idea that IL-8 might be associated with reflux nephropathy and chronic kidney disease [44,51].

However, there are also published studies that present contradictory results, which is why further investigations are needed, as in the case of IL-6 [51].

### 3.4. Antimicrobial Peptides

AMPs are cationic, amphipathic oligopeptides made of 20 to 60 amino acids, representing the most primitive elements of the immune system. Intercalated cells in the kidneys are the main producers of AMPs, but they are also released from the urothelium of the bladder and ureters. These oligopeptides have the ability to rapidly achieve a high concentration on bacterial membranes, inducing their lysis. Some AMPs can reach bacterial cytoplasm, interfering with proteins or DNA synthesis. Moreover, AMPs can also disrupt the energy processes of bacterial cells, stop cell division, bind vital nutrients, and augment opsonization [28,36,54].

Their characteristics make AMPs an attractive subject of study in order to develop new antimicrobial agents for the treatment and prevention of urinary tract infections. Because of their poor stability, poor intestinal absorption, and poor penetration to the urinary tract, the oral administration of AMPs is not a solution to be considered. Therefore, the stimulation of endogenous AMP production is attempted. So far, the administration of oral vitamin D, estrogen, zinc, and short-chain fatty acids seems to be effective in stimulating AMPs’ production [26,28,36].

Lately, AMPs are also studied for their potential value as diagnostic and prognostic biomarkers in urinary tract infections and other renal disorders [36].

This class of immune effectors comprises defensins, cathelicidin, ribonucleases, and metal-binding proteins [28,54].

#### 3.4.1. Cathelicidin

Human LL-37 is an amphipathic, linear oligopeptide that adopts an α-helical structure when in contact with a bacterial cell membrane. It is produced by epithelial cells, myeloid bone marrow cells, neutrophils, monocytes, and natural killer cells. It provides antimicrobial activity against viruses, as well as Gram-negative and Gram-positive pathogens, by lysing bacterial membranes. In addition, LL-37 has the ability to prevent biofilm formation, promote phagocytosis by stimulating neutrophil extracellular trap formation, and act as a chemoattractant for monocytes and neutrophils [54,55].

In healthy subjects with non-infected urine, cathelicidin concentrations are very low. After exposure to a uropathogenic agent, it is quickly synthesized and released within minutes, which makes it an important element of the first line of the innate immune response. In the first phase of infection, LL-37 is produced by urothelial cells; after that, in more advanced stages of infection, neutrophils are the main manufacturer [26,54].

Gene expression of cathelicidin is influenced by vitamin D. This fact is the starting point in trying to influence the susceptibility of individuals to urinary infections. Some studies have shown that children with vitamin D deficiency may not be able to increase urine LL-37 concentrations during infections. Further studies are needed to investigate whether vitamin D supplementation can reduce the recurrence of UTIs [56,57].

#### 3.4.2. Defensins

Along with cathelicidin, defensins are the most studied AMPs. They are highly structured compact oligopeptides formed by 15–10 amino acids, with a wide antimicrobial activity spectrum against viruses, bacteria (Gram positive and negative), fungi, and protozoa. In addition, defensins have chemoattractant properties on immature dendritic cells, playing a role in the cell-mediated immune response. Depending on spatial conformation determined by the disulphide-binding pattern, defensins are classified into α and β subfamilies [26,36,58].

*α defensins* are produced by promyelocytes in the bone marrow and stored in neutrophils, being in charge of the non-oxidative antimicrobial activity of neutrophils. Known also as human neutrophil peptides (HNPs), they are classified into four subcategories, HNP1 to HNP4. HNPs attack pathogens after degranulation on the cell surface or after phagocytosis. Ihi et al. have shown in their studies that HNP1-3 are involved in the immune response in UTIs, their concentration increasing fourfold during acute episodes. Furthermore, Tikhonov et al. have demonstrated that in chronic pyelonephritis, HNP1 concentrations increase eightfold compared to controls [58,59,60].

The urothelium of kidneys, ureters, and the bladder expresses human α defensin 5 (HD5), which seems to have bactericidal activity against UPEC, Enterococcus faecium, Klebsiella pneumoniae, and Pseudomonas aeruginosa. Despite the fact that HD5 is not detected in non-infected urine, its concentrations increase in UTIs, and it is supposed that higher levels are present on the mucosal surface. Published data suggest that HD5 may be used as a new biomarker of UTIs [33,61].

*β defensins* are peptides with antibacterial activity against Gram-negative and Gram-positive bacteria. Human β defensin 1 (HBD1) and human β defensin 2 (HBD2) have been detected in the human urinary system. HBD1 is constitutively expressed in epithelial cells of the loop of Henle, distal tubules, and collecting ducts, and HBD2 is expressed by the same structures of the kidney but only during infections, especially in chronic pyelonephritis [36,58,59].

Urinary levels of HBD1 are not high enough to achieve bactericidal concentrations, but it is assumed that HBD1 forms a protective layer on the surface of the urothelium that prevents uropathogens’ attachment. In acute pyelonephritis, concentrations of HBD1 increase threefold, but without response in cystitis. Moreover, in vitro studies suggest that HBD1 stimulates neutrophil extracellular trap formation [58,61].

In addition to its direct antibacterial activity, HBD2 is involved in the chemotaxis of macrophages, immature dendritic cells, monocytes, granulocytes, and memory T cells [26].

#### 3.4.3. Metal-Binding AMPs

Several AMPs act as bacteriostatic agents by binding certain metals such as iron, nickel, or zinc. The latter represent micronutrients that are mandatory for the growth of microorganisms. Among AMPs with chelating activity, we mention hepcidin, lipocalin, lactoferrin, and calprotectin [36].

*Hepcidin* is a peptide with a role in iron hemostasis, which is synthesized in hepatic cells and excreted in the urine stream. It is also known as liver-expressed antimicrobial peptide-1 (LEAP-1). Hepcidin prevents cellular efflux of iron by attaching to ferroportin (iron export channel). The most common form of hepcidin detected in urine consists of 25 amino acids. Known also as hepc-25, this form has urine concentrations between 10–30 μg/L [26,62]. In addition to its bacteriostatic activity by iron sequestration, Hepcidin also has a direct bactericidal activity, being efficient against *E. coli*, *S. aureus*, group B *Streptococcus*, *S. epidermidis,* and *Candida albicans* [42].

*Lactoferrin* is a multifunctional single-chain glycoprotein excreted in milk, tears, mucus, blood, and saliva. In the kidneys, lactoferrin is expressed in the distal collecting tubules, being present on the luminal surface, with low urine concentrations between 14–145 ng/mL. Its antimicrobial activity is due to iron sequestration and direct bacterial membrane cell damage. Despite lactoferrin’s activity against bacteria, its excretion is not increased after UTIs or renal injury [36,63].

*Lipocalin 2*, also known as neutrophil gelatinase-associated lipocalin (NGAL), is a protein member of the lipocalin superfamily with multiple biological activities. NGAL is a 25 kDa protein consisting of 178 amino acids. First described in granules of neutrophils, NGAL is also present in renal tubular cells, hepatocytes, and cardiomyocytes, being involved in the innate immune response to bacterial infections. It is also expressed in the prostate, uterus, salivary glands, trachea, lungs, stomach, and colon. In healthy individuals, both urinary and plasma concentrations are negligible [36,64].

The main function of NGAL is related to siderophores’ binding. The latter represent small compounds with a high affinity for iron, which are used by certain microorganisms in order to achieve the iron necessary for survival, growth, and replication. Some studies have demonstrated that genetically modified animals with deficiencies in NGAL secretion have been more susceptible to severe *E. coli* infections and have died faster due to sepsis. After bacterial insult, NGAL is immediately secreted in order to provide protection to the urothelium and reduce colonization of the urinary tract with uropathogens. In patients with cystitis, urinary concentrations of NGAL increase tenfold, in correlation with the microbial load. In studies on human subjects, the plasma level of NGAL was an effective marker of differentiation between viral and bacterial infections [54,65,66].

In addition to its implications in infectious diseases, NGAL concentrations in plasma and urine seem to be a sensitive marker for acute and chronic renal injury. NGAL detection in urine precedes other proteins’ appearance, such as β2 microglobulin or N-acetyl-β-d-glucosaminidase, after ischemic or nephrotoxic acute kidney injury (AKI). The studies conducted so far suggest that in addition to its value as an early predictive marker for AKI, NGAL can also be used as a sensitive biomarker for the determination of the severity and progression of CKD. In critically ill patients, NGAL proved to be a valuable indicator of the need for renal replacement therapy and mortality [64,67,68,69].

In one of their studies, Yilmaz A et al. suggest that urinary NGAL levels are higher in patients with renal scars compared with controls [70].

To sum up, NGAL represents a new promising biomarker with high sensitivity in the early detection of UTIs. Moreover, it can be used as a sensible, specific, and early predictor of acute kidney injury and also as a progression biomarker for chronic kidney disease [36].

*Calprotectin*, also known as S100A8/A9, is a heterodimer consisting of peptides S100A8 and S100A9, which is expressed by macrophages, monocytes, and granulocytes. S100A8/A9 present antibacterial activity due to the high affinity in binding manganese and zinc, two important micronutrients for bacterial survival and growth. Even in patients with urosepsis levels of calprotectin increase, the effect of UTIs on urinary calprotectin expression are still unknown [36,71].

Lately, calprotectin was found to be a promising biomarker in differentiation between intrinsic and prerenal AKI. When calprotectin was studied for its benefits in predicting the need for renal replacement therapy, the results have shown a moderate accuracy in comparison with NGAL [36,72].

#### 3.4.4. Ribonuclease

A superfamily consists of several peptides with antimicrobial activity, which are involved in innate immune response, such as ribonucleases 6, 7, and 8, angiogenins, and eosinophil-produced ribonuclease. Even though their antibacterial activity is not completely understood, it has been observed that ribonucleases 6 and 7 are effective agents against the most frequent Gram-negative and Gram-positive pathogens involved in urinary tract infections [73,74].

*Ribonuclease 7* is the most studied member of this family, being one of the most important and potent AMPs involved in preserving human urinary tract sterility. Its baseline urinary concentrations, with ranges higher than those of other AMPS, are enough to ensure bactericidal activity. During acute pyelonephritis, the concentration of RNase 7 doubles as a front-line response against uropathogens. Having a positive charge surface, ribonuclease 7 easily interacts with the bacterial cell surface, which is negatively charged, being thus able to penetrate and disrupt the integrity of the cellular wall, with this mechanism being independent of its ribonuclease activity. RNase 7 activity is regulated by its interaction with ribonuclease inhibitors, as well as expression and activity of the latter, decreasing in acute pyelonephritis [75,76,77].

Whereas RNase 7 is only expressed in primates by the intercalated cells of the renal collecting tubules and the urothelium of the lower urinary tract, RNase 6 is expressed both in murine models and in humans by leukocytes, macrophages, and granulocytes. Unlike ribonuclease 7, ribonuclease 6 is not detectable in non-infected urine, its secretion occurring in acute pyelonephritis, within the first hour after bacterial invasion [76,77].

### 3.5. Antimicrobial Proteins

**Figure 4:**
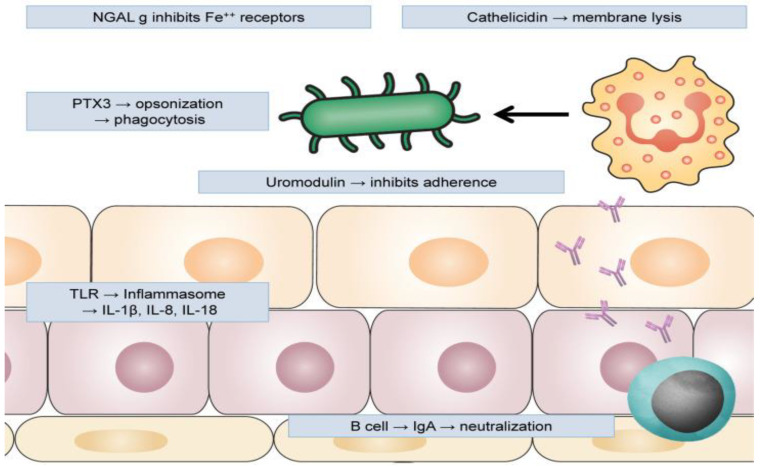
Uromodulin produced by urothelium inhibits adherence of UPEC; NGAL (neutrophil gelatinase-associated lipocalin) inhibits iron receptors, blocking bacterial capacity to proliferate; cathelicidin and other antimicrobial peptides cause membrane lysis; pentraxin 3 (PTX3) opsonizes uropathogens and facilitates their phagocytosis by neutrophils and macrophages; epithelial cells activate inflammasome trough TLRs, producing pro-inflammatory cytokines; adaptive immune response also participates with B lymphocytes, producing antigen-specific IgA-neutralizing uropathogens. (Reproduced with permission from O [26]).

In addition to AMPs, there have been isolated proteins from urine with a role in maintaining sterility of the urinary tract. Such proteins involved in the innate immune system are Tamm–Horsfall protein (THP), also known as Uromodulin, and Pentraxin-related protein 3 (PTX 3) (Figure 4) [26].

#### 3.5.1. Uromodulin

Tamm–Horsfall protein is a glycoprotein with polymeric structure that is present in high concentrations in urine (50–150 mg/day), being produced by the epithelial cells of the loop of Henle. In the form of aggregate THP, it can reach molecular weights up to one million Da. Under the action of some substances such as urea, acetic acid, guanidine, or sodium dodecyl sulfate, Uromodulin aggregates decompose into monomers with a molecular weight of 95 kDa. Due to the ratio between acidic and basic amino acids in its structure, Uromodulin has low solubility in water and neutral solutions. After dissolution in alkaline media, THP tends to take the form of a gelatinous substance, which makes it an important component of the structure of hyaline casts that may be present in the urine. In addition to its capacity to be used as a biomarker for investigation of the renal tubules, THP also has a role in normal function of the thick ascending limb of the loop of Henle, by being involved in electrolytes’ reabsorption. Moreover, in the urinary tract, Uromodulin plays a protective role due to its ability to bind uropathogens, endogenous harmful molecules such as Sd antigen, lectin-like molecules, and the extracellular matrix [78,79,80].

Tamm–Horsfall protein is involved in pathological conditions of the reno-urinary tract associated with inflammatory response, such as nephrolithiasis, tubulointerstitial nephritis, and pyelonephritis [81].

Depending on the injured renal structure and the moment of production, Uromodulin may have different functions. For example, it can suppress neutrophil activity and infiltration, resulting in tubular injury resolution. By suppressing local production of IL-23 and IL-17, it plays an important role in granulopoiesis inhibition. On the other hand, Uromodulin is able to increase renal inflammation response by stimulating dendritic cell activation in conditions associated with interstitial injury [79,81].

Uromodulin is a protein with a proven role in the homeostasis of the immune system, both at the systemic level and at the level of the urinary system. The spatial conformation, on the one hand, and the ionic charge, on the other hand, favor THP interaction with proinflammatory cytokines, such as TNFα, IL1β, and IL2, and with the c1 and c1q fractions of the complement. In this way, THP becomes a trap for proinflammatory cytokines and inhibits the classical pathway of complement activation [78,79,80].

THP is also found in large amounts in amniotic fluid, exerting an immunosuppressive effect on T cells that prevents the allogeneic rejection of the fetus. Other immunomodulatory effects exerted by THP occur secondary to the adhesion of this protein to the surface of certain cells present in the bloodstream. Thus, after adhesion to the surface of polymorphonuclear leukocytes, THP stimulates phagocytosis; after adhesion to the surface of lymphocytes, it stimulates their proliferation; and after adhesion to the surface of monocytes or macrophages, it enhances phagocyte activation. By interacting with peripheral blood mononuclear cells, THP promotes, depending on the dose, the production of TNFα, IL1β, IL6, and superoxide anion radicals [79,80,81].

As an immunomodulatory agent, it has been observed that THP induces a stimulating effect on naive immune cells and an inhibitory effect on active immune cells (lymphocyte proliferation). Thus, THP has the ability to protect the urinary system from excessive damage of the renal parenchyma after a bacterial infection due to its capacity to bind cytokines and due to its influence on pro- and anti-inflammatory cytokines’ production by peripheral blood mononuclear cells [82,83,84].

Tamm–Horsfall protein also has a crucial role in maintaining urine sterility. It does not present direct bactericidal activity, but it prevents bacterial colonization in the urinary tract by inhibiting the adhesion of uropathogens to the urothelium. THP adopts a specific 3D structure with pores in which uropathogens are trapped, thus being subsequently eliminated from the urinary tract through the urine stream. Uromodulin deficiency leads to intense bacterial colonization, chronic inflammation of the bladder, and prolonged recovery time after an infection [85,86].

In acute urinary infections, antibodies against Uromodulin are synthesized. The target of these antibodies is the complex formed by bacteria bounded by Uromodulin, which is subsequently eliminated by neutrophils through adhesion and phagocytosis [80].

Impairment of the genetic expression of uromodulin results in certain disorders, such as progressive dysfunction of the distal tubule, urinary lithiasis, salt-sensitive arterial hypertension, juvenile hyperuricemic nephropathy, and renal damage [82,87].

In several conditions such as lupus nephritis, diabetic nephropathy, and chronic renal disease, THP production is decreased, which increases the susceptibility to urinary tract infections. Moreover, decreased urinary concentrations of uromodulin may predict renal tubular dysfunction regardless of etiology. Low urinary uromodulin production may also accelerate the inflammatory process in the kidneys in certain chronic diseases as a result of decreased clearance of proinflammatory cytokines [80,81,86].

In certain diseases in which extensive lesions of the renal tubules occur, Uromodulin may be deposited in the renal interstitium, where an inflammatory process mediated by polymorphonuclear leukocytes and mononuclear cells will be initiated, resulting in interstitial inflammation and anti-Uromodulin antibodies’ production. Vesicoureteral reflux, repeated urinary infections, obstructive uropathies, medullary cystic disease, endemic nephropathy, and renal transplant rejection are in this category of pathological conditions in which these antibodies are found. These observations suggest that the dosing of Uromodulin and anti-Uromodulin antibodies from urine can be used as a prognostic biomarker for the development of interstitial nephritis in these diseases [80,84].

Uromodulin plasma concentrations are directly related to urinary concentrations, but with significant lower values (70–540 ng/mL). In patients with chronic kidney disease, THP plasma concentrations are extremely low and correlate with creatinine clearance in people with chronic kidney disease, so this can be a useful tool for the early identification of chronic kidney disease. After kidney removal, serum concentrations become undetectable, but with increasing values after kidney transplantation. This increase in concentration is correlated with the uptake of function by the renal graft. Moreover, serum THP levels may predict renal graft dysfunction, similar to creatinine levels, creatinine clearance, cystatin C, or blood urea nitrogen levels. The same behavior of THP concentrations was observed among pediatric patients. In healthy individuals, THP levels increase with age, and they decrease in correlation with creatinine clearance in patients with chronic kidney disease. In pediatric patients with vesicoureteral reflux and obstructive uropathies, Uromodulin levels are higher, even in patients with declining creatinine clearance [79,80,83,88].

#### 3.5.2. Pentraxin 3

**Figure 5:**
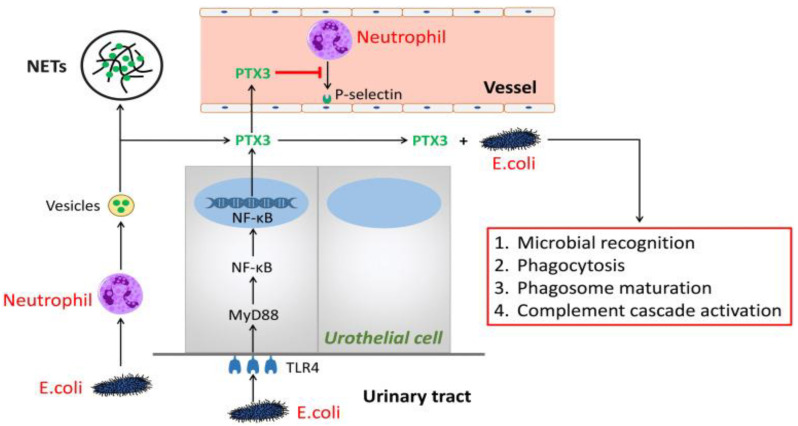
The role of PTX3 in urinary tract infection. In the early phase of urinary tract infection, *E. coli* stimulates neutrophil-releasing vesicles that enter the extracellular environment and the release of PTX3. One part of PTX3 participates in constructing extracellular DNA fibrillary networks. Additional part of PTX3 inhibits neutrophil-binding P-selectin and combines with *E. coli* to promote the processes of microbial recognition, phagocytosis, and phagosome maturation, as well as to complement cascade activation. PTX3, which is produced by the urothelial cells via the TLR4/MyD88/NF-κB-signaling pathway, also does so. (Reproduced with permission from [89]).

Pentraxin 3 represents an acute-phase protein from the family of pentraxins, which is involved in inflammatory reaction and the innate immune system response, with a role in immunologic tolerance, complement activation, the opsonization of pathogens, and the removal of apoptotic cells (Figure 5). They are also involved in angiogenesis and tissue remodeling. The pentraxin family contains two types of proteins, namely, short pentraxins and long pentraxins. The first group includes serum amyloid P (SAP) and C-reactive protein (CRP), which are synthesized in the liver, being used as biomarkers of systemic inflammation. On the other hand, pentraxin 3 (PTX3) is included in the group of long pentraxins. It is synthesized locally at the site of inflammation by neutrophils, macrophages, monocytes, fibroblasts, dendritic cells, and activated endothelial cells. In the kidneys, PTX3 is produced by mesangial cells, renal fibroblasts, and tubular epithelial cells. The feature of being produced and released at the site of the inflammatory process, under the influence of proinflammatory cytokines (such as IL1 or TNFα), makes PTX a biomarker that correlates with the activity and severity of the disease [36,89,90].

In physiological conditions, pentraxins are present in very low concentrations or are even undetectable, with increasing plasma concentrations in certain diseases associated with systemic inflammatory response, such as septic shock, myocardial infarction, autoimmune diseases (psoriasis, rheumatoid arthritis, small vessel vasculitis), and atherosclerosis [89,90].

As mentioned above, PTX3 plays a role in the innate immune response during viral, bacterial, or fungal infections. This also happens in urinary tract infections. Whether it is cystitis or pyelonephritis, urinary concentrations of PTX3 increase in correlation with the severity of the infection. In murine models with PTX3 deficiency, it is also shown that PTX3 is important in the natural defense against uropathogens, and this correlates with persistent inflammation and kidney damage. This is why it is believed that PTX3 plays a role in the pathophysiology of renal parenchyma damage secondary to persistent inflammation. In patients without urinary tract infections and without impaired renal function, it was observed that high concentrations of PTX3 in urine are correlated with the presence of renal scars. This observation may be the starting point in using PTX3 as a reliable biomarker of parenchymal damage, secondary to vesicoureteral reflux or after recurrent urinary infections. However, there are also several other conditions in which urinary PTX3 levels are increased, such as membranoproliferative glomerulonephritis, IgA nephropathy, lupus nephritis, focal segmental glomerulosclerosis, diabetic nephropathy, and Henoch–Schönlein purpura nephritis [90,91,92,93,94].

Another observation with possible implications in clinical management in patients with kidney disease is that TXP3 concentrations are associated with lower creatinine clearance. Moreover, TXP3 levels start to increase before impaired renal function, which represents an early marker of renal tissue damage [95,96].

In patients with kidney transplant and acute allograft rejection, an up-regulated expression of TXP3 has been observed, which correlates with the severity of dysfunction and histological modifications. This suggests the possibility of its use as a biomarker in acute renal allograft rejection [92,96].

## 4. Discussion

The innate immune system of the urinary tract represents the first line of defense against uropathogens, being able to trigger a response immediately after the bacterial invasion. After colonization and ascension in the urinary tract, uropathogens are restrained to adhere to the urothelium by Tamm–Horsfall protein and by the fluid layer adjacent to the epithelium, consisting of defensins and cathelicidin. If these first mechanisms are overwhelmed, and the bacteria make contact with epithelial cells, toll-like receptors are activated. Subsequently, interleukins are synthesized, and AMPs are released in the urinary stream, thus initiating the inflammatory response. Due to the chemoattractant properties of AMPs and interleukins, cellular effectors of the innate immune response are recruited at the site of invasion. In turn, these cells will stimulate the production of interleukins and AMPs, amplifying the response against bacteria. If this response is not effective in bacterial clearance, the inflammatory process that is triggered may cause tissue damage and, if persistent, may lead to fibrosis and the development of renal scars [19,27,28].

On the other hand, renal diseases that evolve with the alteration of the architecture and the function of the renal structures compromise the physiological production of interleukins, AMPs, and other protective proteins. As a result, the immune response is inefficient and even harmful, leading to increased susceptibility to UTIs and the development of chronic kidney disease. Therefore, patients with chronic conditions such as VUR, obstructive nephropathy, renal dysplasia, and polycystic disease have recurrent UTIs. In these cases, modulation of the immune mechanisms could be an efficient therapeutic option [36,51].

In the context of increasing bacterial resistance to antibiotics, augmentation of the innate immune response could be an alternative in preventing and treating urinary tract infections in children susceptible to frequent recurrences. In this regard, the administration of certain hormones and vitamins has been shown to be effective in stimulating the production of AMPs. Thus, 25-hydroxycholecalciferol (25(OH)-D3) and 1,25-dihydroxycholecalciferol (1,25(OH)2-D3) stimulate antimicrobial activity by increasing the production of LL-37. Furthermore, it has been observed that oestrogen administration has the ability to increase defensins and RNase production. In addition to stimulating natural synthesis, oral, intravenous, or local administration of AMPs could bring major benefits in the management of patients with recurrent UTIs. An important aspect to note is that bacteria do not develop mechanisms of resistance to AMPs, and this is why the latter are a potential alternative in the prevention and treatment of UTIs [24,54].

As mentioned above, some of the peptides involved in the innate immune response have, in addition to antimicrobial activity, a role in persistent inflammation and fibrogenesis in the renal parenchyma. Therefore, they can be used as diagnostic and prognostic biomarkers in UTIs, uropathies, reflux nephropathy, or in chronic kidney disease. In this regard, urinary IL-8 concentrations are proven to be higher in patients with VUR and when renal scarring occurs. IL-8/creatinine in urine seems to be an accurate tool in VUR diagnostics. Moreover, IL-8 levels could be correlated with the degree of VUR and could predict the evolution to RN. Increased urinary IL-6/creatinine in the acute phase of UTIs is correlated with renal scar occurrence on long-term follow-up. The elevated serum concentration of the soluble IL-2 receptor was interpreted as an efficient predictor of progression to CKD in individuals with VUR. In addition, serum levels of IL-6, TNFα, IL-1b, and TGFβ1 were correlated in some studies with the presence of VUR and RN, which indicate them as possible diagnostic and prognostic biomarkers. Other studies suggest that MCP1, TGFβ1, and VEGF may be useful in the diagnosis and monitoring of renal parenchymal fibrosis in patients with VUR. One more noninvasive marker in the diagnosis and prediction of renal scarring in children with VUR seems to be urinary NGAL [50,97,98].

## 5. Conclusions

Improvement of the clinical management of patients with reno-urinary pathology and a better understanding of the innate immune mechanisms involving the urinary tract are needed. Therefore, further studies are required to complete current knowledge and to promote new research directions regarding diagnostic and therapeutic tools in renal pathology.

## Data Availability

No new data were created or analyzed in this study. Data sharing is not applicable to this article.
